# Prevalence of malnutrition in patients at first medical oncology visit: the PreMiO study

**DOI:** 10.18632/oncotarget.20168

**Published:** 2017-08-10

**Authors:** Maurizio Muscaritoli, Simone Lucia, Alessio Farcomeni, Vito Lorusso, Valeria Saracino, Carlo Barone, Francesca Plastino, Stefania Gori, Roberto Magarotto, Giacomo Carteni, Bruno Chiurazzi, Ida Pavese, Luca Marchetti, Vittorina Zagonel, Eleonora Bergo, Giuseppe Tonini, Marco Imperatori, Carmelo Iacono, Luigi Maiorana, Carmine Pinto, Daniela Rubino, Luigi Cavanna, Roberto Di Cicilia, Teresa Gamucci, Silvia Quadrini, Salvatore Palazzo, Stefano Minardi, Marco Merlano, Giuseppe Colucci, Paolo Marchetti, on behalf of the PreMiO Study Group

**Affiliations:** ^1^ Department of Clinical Medicine, Sapienza University of Rome, Rome, Italy; ^2^ Department of Public Health and Infectious Diseases, Sapienza University of Rome, Rome, Italy; ^3^ Department of Medical Oncology, National Cancer Research Centre Giovanni Paolo II, Bari, Italy; ^4^ Department of Medical Oncology, Catholic University of Sacred Heart, Largo A. Gemelli, Rome, Italy; ^5^ Medical Oncology Unit, Ospedale Sacro Cuore Don Calabria, Verona, Italy; ^6^ Oncology Unit, Antonio Cardarelli Hospital, Naples, Italy; ^7^ Oncology Unit, San Pietro Fatebenefratelli Hospital, Rome, Italy; ^8^ Department of Clinical and Experimental Oncology, Medical Oncology 1, Veneto Institute of Oncology IOV - IRCCS, Padua, Italy; ^9^ Department of Oncology, University Campus Bio-Medico of Rome, Rome, Italy; ^10^ Department of Medical Oncology, Azienda Ospedaliera Civile - Maria Paternò Arezzo, Ragusa, Italy; ^11^ Medical Oncology, Clinical Cancer Centre, IRCCS-Arcispedale S. Maria Nuova, Reggio Emilia, Italy; ^12^ Department of Oncology-Hematology, Guglielmo da Saliceto Hospital, Piacenza, Italy; ^13^ Medical Oncology Unit, S.S. Trinita Hospital, Sora, Italy; ^14^ Division of Medical Oncology, Mariano Santo Hospital, Azienda Ospedaliera, Cosenza, Italy; ^15^ Medical Oncology, Oncology Department, S. Croce & Carle Teaching Hospital, Cuneo, Italy; ^16^ Medical Oncology Department, National Cancer Research Centre Giovanni Paolo II, Bari, Italy; ^17^ Department of Clinical and Molecular Medicine, Faculty of Medicine and Psychology Sapienza, St. Andrea Hospital, Rome, Italy; ^18^ IDI-IRCCS, Rome, Italy; ^19^ The PreMiO Study group also included investigators who contributed to patients’ enrollment

**Keywords:** malnutrition, cancer, cachexia, sarcopenia, oncology

## Abstract

**Background:**

In cancer patients, malnutrition is associated with treatment toxicity, complications, reduced physical functioning, and decreased survival. The Prevalence of Malnutrition in Oncology (PreMiO) study identified malnutrition or its risk among cancer patients making their first medical oncology visit. Innovatively, oncologists, not nutritionists, evaluated the nutritional status of the patients in this study.

**Methods:**

PreMiO was a prospective, observational study conducted at 22 medical oncology centers across Italy. For inclusion, adult patients (>18 years) had a solid tumor diagnosis, were treatment-naive, and had a life expectancy >3 months. Malnutrition was identified by the Mini Nutritional Assessment (MNA), appetite status with a visual analog scale (VAS), and appetite loss with a modified version of Anorexia-Cachexia Subscale (AC/S-12) of the Functional Assessment of Anorexia-Cachexia Therapy (FAACT).

**Findings:**

Of patients enrolled (*N=*1,952), 51% had nutritional impairment; 9% were overtly malnourished, and 43% were at risk for malnutrition. Severity of malnutrition was positively correlated with the stage of cancer. Over 40% of patients were experiencing anorexia, as reported in the VAS and FAACT questionnaire. During the prior six months, 64% of patients lost weight (1–10 kg).

**Interpretation:**

Malnutrition, anorexia, and weight loss are common in cancer patients, even at their first visit to a medical oncology center.

## INTRODUCTION

### Prevalence and consequences of malnutrition in cancer

The high prevalence of cancer-related malnutrition and its negative consequences are taken too lightly in most oncology units. Studies from Germany [1], France [2–4], Spain [5], and Brazil [6] reported malnutrition prevalence ranging from 25% to over 70% based on nutritional assessments. Indeed, people with cancer are among the most malnourished of all patient groups [7]. Malnutrition in cancer patients seems to be particularly evident when a precise measurement of body composition is used for detection (e.g., computerized tomography); in such studies, 50% to 80% of patients had low lean body mass, a correlate of malnutrition [7]. Unfortunately, clinicians often miss malnutrition risk in cancer patients [2], as do many patients and their caregivers [8]. Even when malnutrition risk is recognized, it may not be adequately addressed. Hospital studies in Europe showed that only 1 in 3 cancer patients at risk of malnutrition in fact received nutritional support [3, 5].

When cancer-related malnutrition goes untreated, consequences can be serious. Malnourished colorectal cancer patients tolerated fewer cycles of chemotherapy [9], while other cancer patients with sarcopenia/malnourishment were at high risk for toxicity of chemotherapy [10]. Further, malnourished patients undergoing treatment for oral cancers scored lower on quality of life (QOL) scales related to physical function, while those who were nourished and able to maintain or gain weight had significantly better QOL [11]. Malnutrition also increased financial costs for managing cancer patients, including costs for longer hospital stays and higher rates of complications following cancer-related surgery [4, 5, 12]. At its most severe, patients who were malnourished had a 2- to 5-fold higher risk of dying compared to patients with little or no evidence of malnutrition, as seen in both short- and long-term follow-up studies [1, 4, 9].

Due to generally low awareness of cancer-associated malnutrition, strategies for taking early actions to prevent and treat anorexia, cachexia, and sarcopenia are overlooked by many oncologists. The PreMiO study was conceived to quantify malnutrition and its signs among cancer patients making their first visit for medical oncology care in Italy. Our ultimate goal is to raise oncologists’ awareness to the pressing need for early assessment of nutritional status in cancer patients and the need for providing appropriate nutritional care.

### Definition of malnutrition in cancer

**Figure 1:**
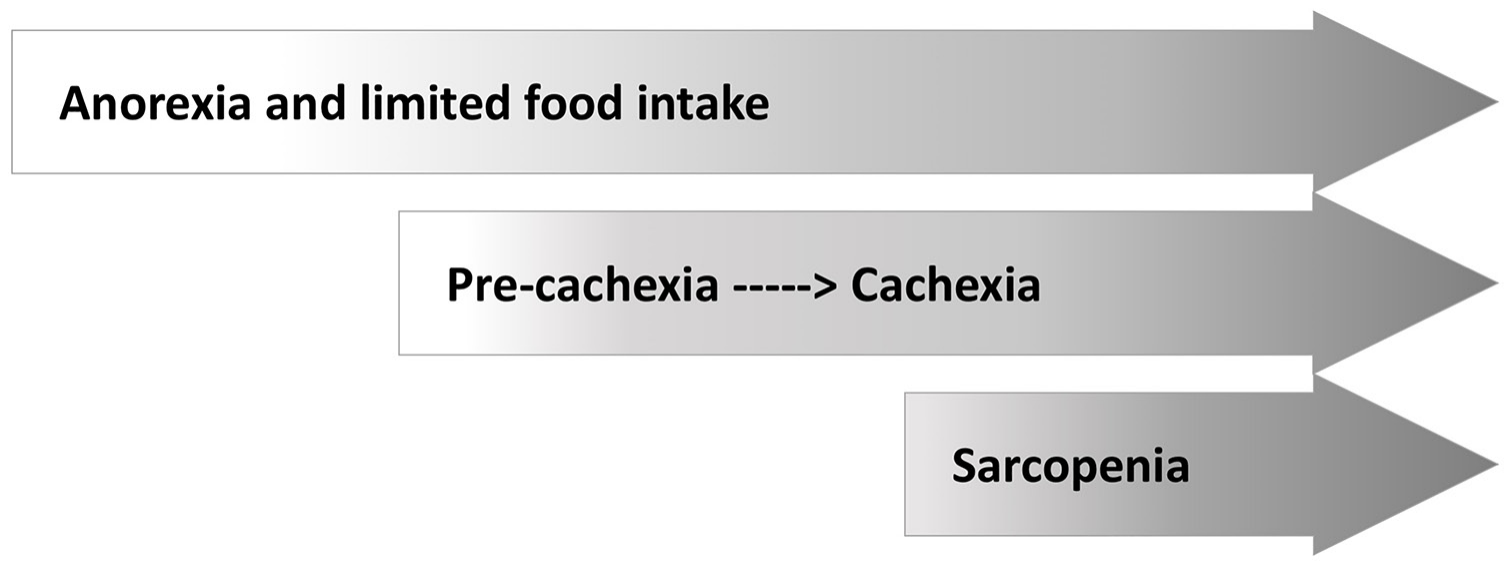
Causes and consequences of malnutrition in cancer: anorexia, cachexia, and sarcopenia

Malnutrition in cancer patients differs dramatically from malnutrition due to simple starvation [13]. The multiple causes and serious consequences of disease-associated malnutrition in cancer include anorexia, cachexia (ranging from pre-cachexia to cachexia), and sarcopenia (Figure 1) [14, 15]. Malnutrition in cancer is a result of inadequate nutritional intake that can lead to a depletion of body stores of fat and lean mass, and ultimately result in reduced physical function [16]. Initially, people with cancer may experience appetite loss resulting from altered appetite signals [17]. Cancer patients may also have physical restrictions that reduce food intake and nutrient absorption such as mouth ulcers, diarrhea, vomiting, pain, intestinal obstructions, or malabsorption [18, 19].

Systemic inflammation is often present in cancer, resulting from pro-inflammatory cytokines released from tumors or immune cells [7]. This inflammation may increase the body’s metabolic needs, depress appetite, and initiate accelerated muscle protein catabolism. Cachexia, the resulting multifactor wasting syndrome, extends across a spectrum from pre-cachexia (identifiable by clinical symptoms and metabolic markers) to extensive weight loss in refractory cachexia [7, 10, 14, 15, 20].

When anorexia and cachexia/inflammation continue to progress, muscle mass can become depleted, causing the typical cachectic phenotype of the last stages of disease. The loss of lean body mass, and resulting loss of physical function, is known as sarcopenia. Muscle wasting and sarcopenia also occurs in overweight and obese patients, undermining their physical function while retaining an appearance of obesity. This makes sarcopenia particularly difficult to detect in the growing population of overweight and obese cancer patients [7, 10]. Notably with cancer, the loss of skeletal muscle is a powerful negative prognostic factor for people of any body mass index (BMI) [21]. Skeletal muscle mass loss is associated with higher risk of toxicity from chemotherapy, reduced time to tumor progression, poor surgical outcome, physical function impairment, and increased mortality [21–25].

## RESULTS

### Patient demographics and tumor characterization

Between June 2012 and November 2014, a total of 1952 cancer patients (931 men and 1021 women) were enrolled into the study on their first visit to a medical oncology center. Those enrolled were well distributed among sites in the northern (*N=*651), central (*N=*596), and southern (*N=*705) regions of Italy. Mean age (years) was 62.7±12.9, mean BMI (kg/m^2^) was 24.8±4.4, and mean weight (kg) was 68.4±13.2.

With primary tumor stratification by site, breast cancer was the most frequent, followed by genitourinary tract, colorectal, and lung cancers (Table 1).

**Table 1:** Frequency of primary tumor types with distribution by tumor stage

Primary tumor type	Frequency, % of all tumors	Stage I, %	Stage II, %	Stage III, %	Stage IV, %
**Breast**	22.1	27.5	29.2	16.0	18.5
**Genitourinary tract**	17.7	15.1	15.7	20.6	40.9
**Colorectal**	16.3	4.1	11.0	29.2	50.9
**Lung**	16.0	1.3	3.8	15.3	75.1
**Other cancer**^1^	7.2	17.0	6.4	10.6	41.8
**Gastroesophageal**	6.5	7.1	4.8	15.9	64.3
**Pancreatic**	4.8	0.0	4.3	18.3	67.7
**Head and neck**	3.2	3.2	6.5	25.8	54.8
**Other GI**	3.1	3.3	1.6	19.7	62.3
**Liver/bile duct**	1.8	5.6	0.0	8.3	80.6
**Unknown primary site**	1.3	0.0	0.0	4.0	56.0
**ALL CANCERS**	100	11.6	12.9	18.7	48.0

^1^Other cancer includes: sarcoma, mesothelioma, mesenchymal, skin, endocrine and hematologic tumors.

By cancer stage, 24.5% of all tumors were localized (i.e., stage I and II), 18.7% regionally spread (i.e., stage III), and 48.0% were metastatic (stage IV); tumor stage was not defined in 173 patients (8.9%).

Tumor stages at first oncology visit varied considerably by primary tumor type (Table 1). For this reason, further data analyses were performed by stratifying patients into non-metastatic (M0, *N=*843, cancer stages I to III) and metastatic (M1, *N=*936, cancer stage IV) subgroups.

### Nutritional status: malnutrition, weight loss, appetite loss (anorexia)

**Figure 2:**
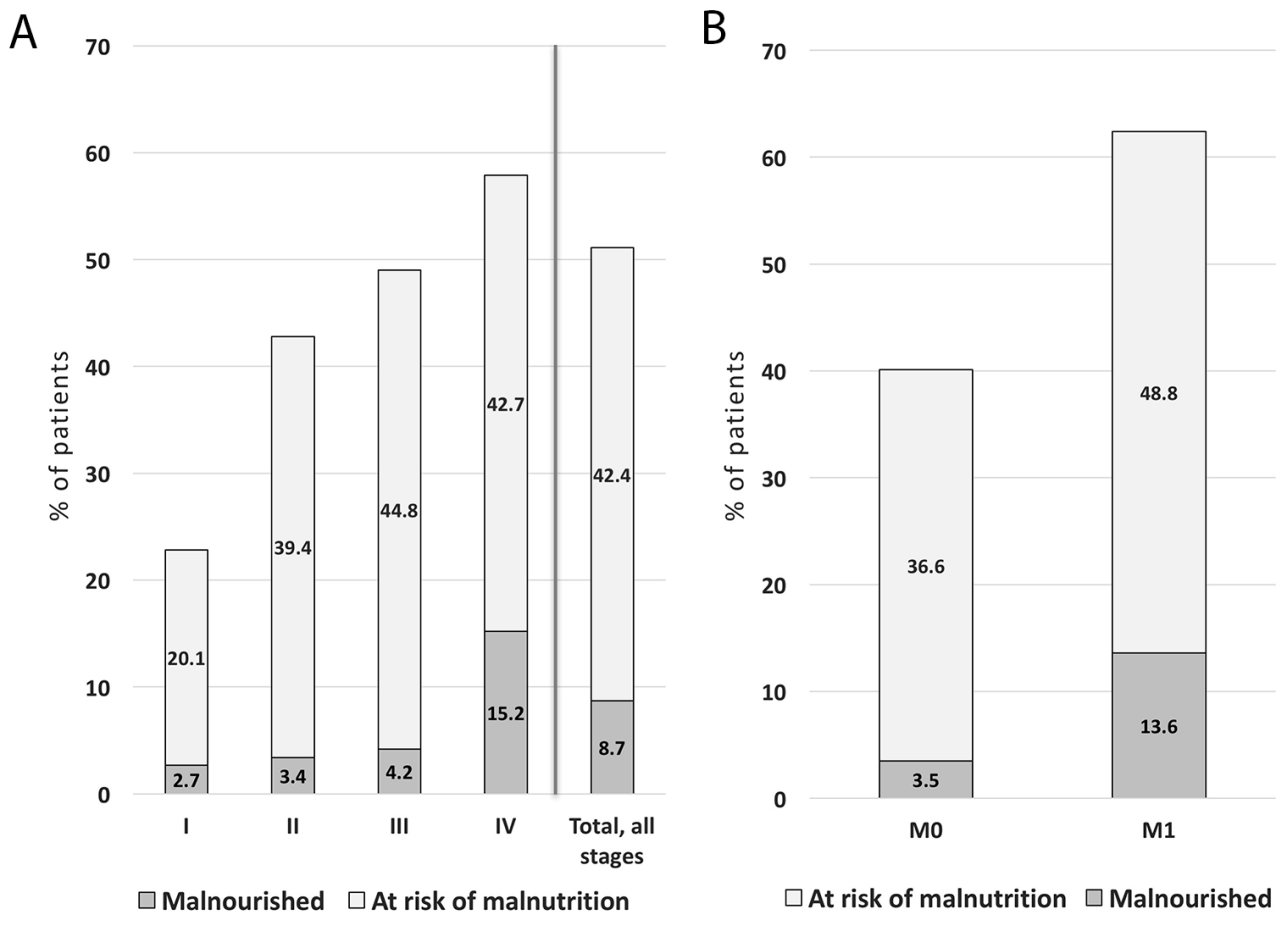
PreMiO patients with malnutrition or malnutrition risk using MNA scoring with results shown by tumor stage and for all tumors **(A)** as well as classified in M0 and M1 groups **(B)** (*N*=1925). *P<*0.001 among cancer stage groups. Malnutrition was defined as MNA score <17, while risk of malnutrition was represented by MNA scores of 17 to 23.5. M0 = stage I-III, M1 = stage IV.

Based on MNA scoring, 51.1% of patients (49.2% of women and 52.9% of men) had nutritional impairment, including risk of malnutrition and overt malnutrition (Figure 2A). Malnutrition and its risk were significantly higher in M1 patients (Figure 2B), (*P<*0.001). As determined by MNA, 40.1% of patients without metastases (M0) already had poor nutritional status at their first oncology visit. Of these, 36.5% were at risk of malnutrition, and 3.5 % were malnourished. Malnutrition was evidenced in 13.6% (*N=*127) of M1 patients.

**Figure 3:**
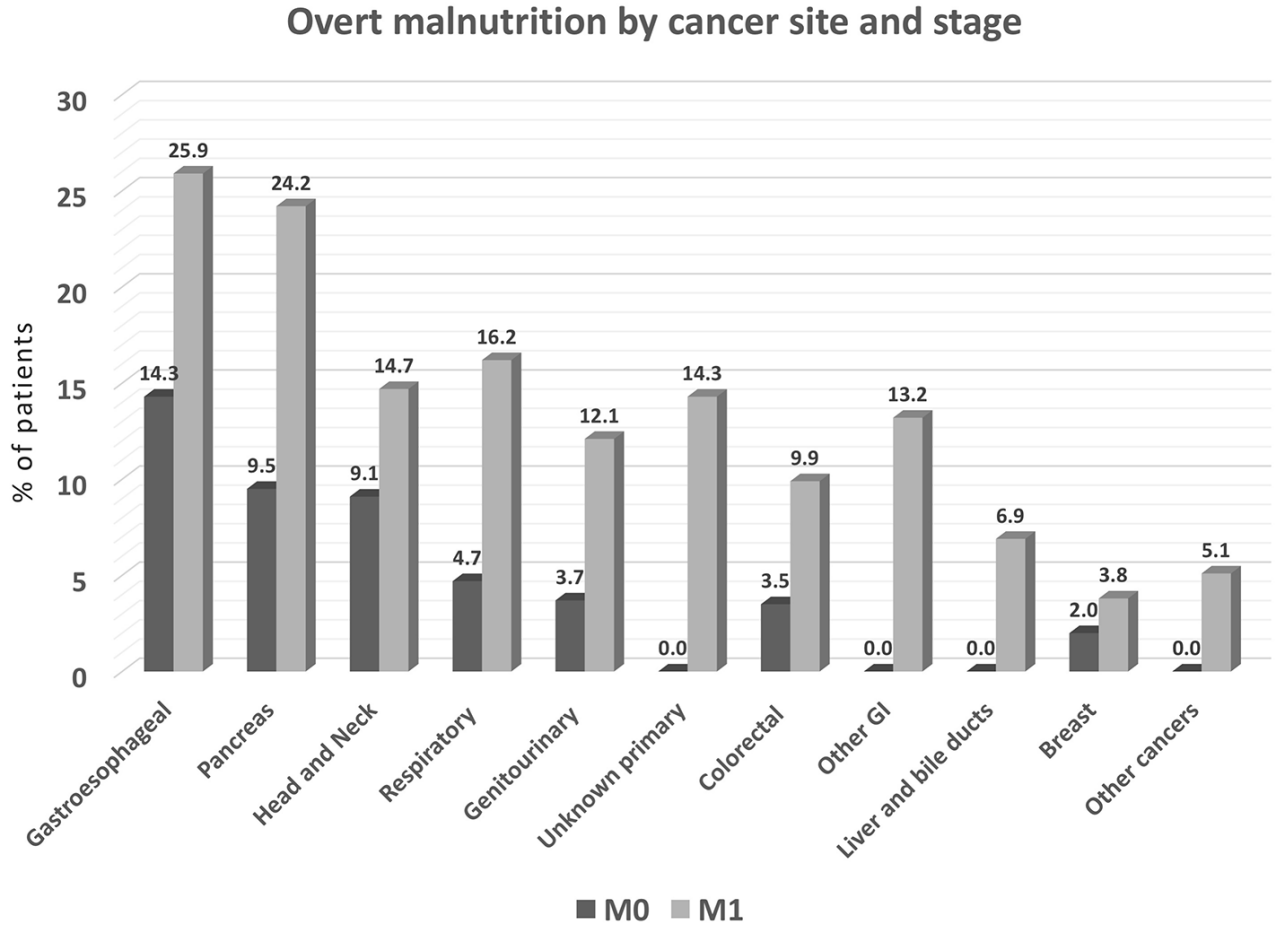
Prevalence of overt malnutrition by cancer site (% of patients with specified tumor type), with malnutrition defined as MNA score <17 (*N=*1925) M0 = stage I-III, M1 = stage IV. *P<*0.001 among cancer site groups.

By site of primary tumor, patients with the highest frequency of malnutrition/undernutrition qualifying MNA score (<17) were those with gastroesophageal, pancreatic, head and neck, and lung tumors (Figure 3); those with breast tumors were least likely to be malnourished. Prevalence of overt malnutrition was significantly higher in M1 compared to M0 patients (*P<*0.001).

Unintentional weight loss is another marker of undernutrition or its risk. At the first medical oncology visit, 65% of patients (*N=*1253) in the study population had experienced weight loss during the previous 6 months: 28.4% lost more than 10% of body weight, 36.2% lost 5% to 10%, and 35.4% lost <5%. The actual weight loss ranged from 1 to 10 kg. Prevalence of unintentional weight loss was greater in M1 patients (76.2%) (*P<*0.001, IC 95% 18.4 to 27.2). Notably, 53% of patients without metastases (M0) had already experienced weight loss at the first oncology visit.

#### Appetite loss/anorexia

Nearly all enrolled patients completed the FAACT questionnaire for anorexia (*N=*1949) and the VAS self-scoring of appetite (*N=*1857). Based on FAACT scores, poor appetite was present in 41% of patients (*N=*802), with mean scores varying by tumor type and stage of disease (Table 2). According to FAACT score, gastroesophageal and pancreatic cancer patients were already anorectic in the non-metastatic phase of the disease. By contrast, all metastatic patients were anorectic based on the FAACT questionnaire (Table 2). By VAS scoring, 44.5% of patients (*N=*826) perceived appetite impairment (VAS score ≤70); the mean VAS score was 67.0±22.6; scores varied by tumor type and disease stage (Table 2). Patients with gastroesophageal, pancreatic and other GI cancer were already anorectic in the non-metastatic phase of the disease. All patients in M1 stage were anorectic based on the VAS (Table 2). Patients with appetite loss reported the main reasons for decreased food intake were early satiety (69%), taste changes (40.3%), nausea or vomiting (31.9%), meat aversion (28.9%) and smell disturbances (16.8%).

**Table 2:** Patient appetite scores by cancer site, based on FAACT (*N=*1949) and VAS scores (*N=*1857)

Cancer site	FAACT M0	FAACT M1	FAACT Total	VAS appetite M0	VAS appetite M1	VAS appetite total
**Breast**	33 ±5	29 ±5	32 ±5	73 ±20	69 ±19	73 ±20
**Genitourinary tract**	32 ±5	28 ±6	30 ±6	72 ±18	61 ±22	67 ±21
**Colorectal**	32 ±5	29 ±5	30 ±5	72 ±23	65 ±22	68 ±22
**Lung**	31 ±5	29 ±5	29 ±6	71 ±24	64 ±23	66 ±23
**Other cancer**^1^	33 ±6	29 ±6	32 ±6	78 ±22	69 ±21	75 ±22
**Gastroesophageal**	27 ±6	23 ±6	25 ±6	58 ±23	52 ±21	54 ±21
**Pancreatic**	28 ±4	24 ±7	25 ±6	62 ±22	48 ±27	53 ±26
**Other GI**	34 ±5	28 ±6	30 ±5	69 ±18	62 ±21	63 ±21
**Liver/bile duct**	33 ±2	26 ±5	28 ±5	82 ±8	62 ±21	65 ±20
**Head and neck**	33 ±6	30 ±5	31 ±5	75 ±23	64 ±19	68 ±22
**Unknown primary site**^2^	26	25±6	28 ±6	20	45 ±17	55 ±24
**ALL CANCERS**	32 ±5	28 ±6	30 ±6	72 ±21	62 ±23	67 ±23

Cutoff points for poor appetite are FAACT ≤30 and VAS appetite score VAS ≤70. M0 = stage I-III, M1 = stage IV. Data are expressed as Mean ±SD

^1^Other cancer includes: sarcoma, mesothelioma, mesenchymal, skin, endocrine and hematologic tumors.

^2^ N=1 if SD not indicated.

FAACT, Functional Assessment of Anorexia-Cachexia Therapy (questionnaire); VAS, visual analog scale of appetite SD, standard deviation.

Patients who assessed their disease as “severe and difficult to cure” had a lower appetite based on FAACT and VAS scores, and higher degree of malnutrition according to MNA (*P<*0.001; Supplementary Tables 1 –3).

### Cachexia and pre-cachexia

**Figure 4:**
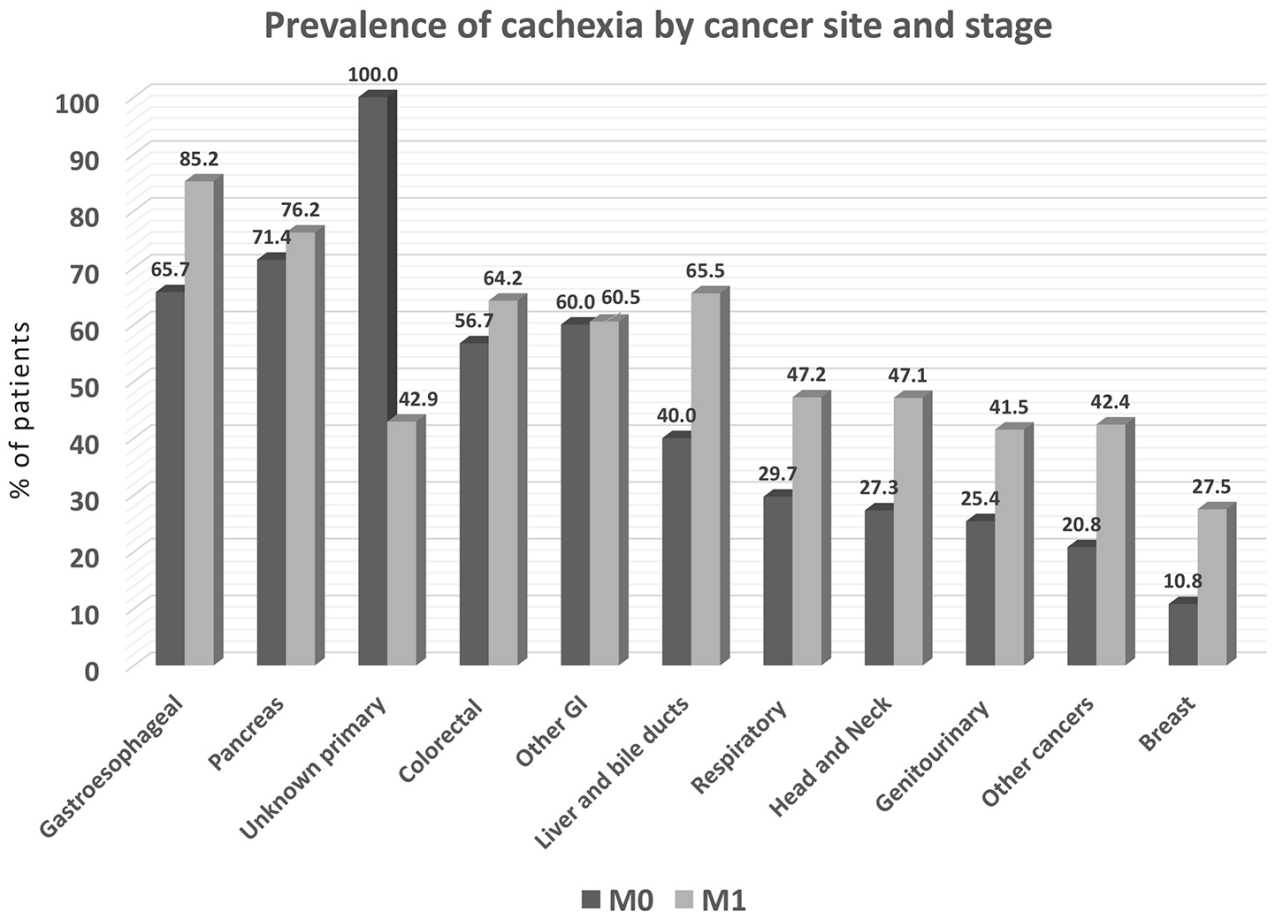
Prevalence of cachexia by primary tumor type in the study population (*N=*1952) Cachexia is defined by weight loss >5% or by the dual criteria of BMI <20 with weight loss of 2% to 5%. M0 = stage I-III, M1 = stage IV. *P<*0.001 among cancer site groups.

An unexpectedly high proportion of patients met the criteria for cachexia in both M0 and in M1 groups. More than 70% of pancreatic and gastroesophageal cancer patients, more than 60% of liver, colorectal, and GI tract, and more than 40% of lung, head and neck, and genitourinary cancer patients could be classified as cachectic, based on BMI and weight loss (Figure 4) using the criterion based on Fearon et al [14]. A notably high proportion of M0 patients exhibited cachexia, including those with breast cancer.

**Figure 5:**
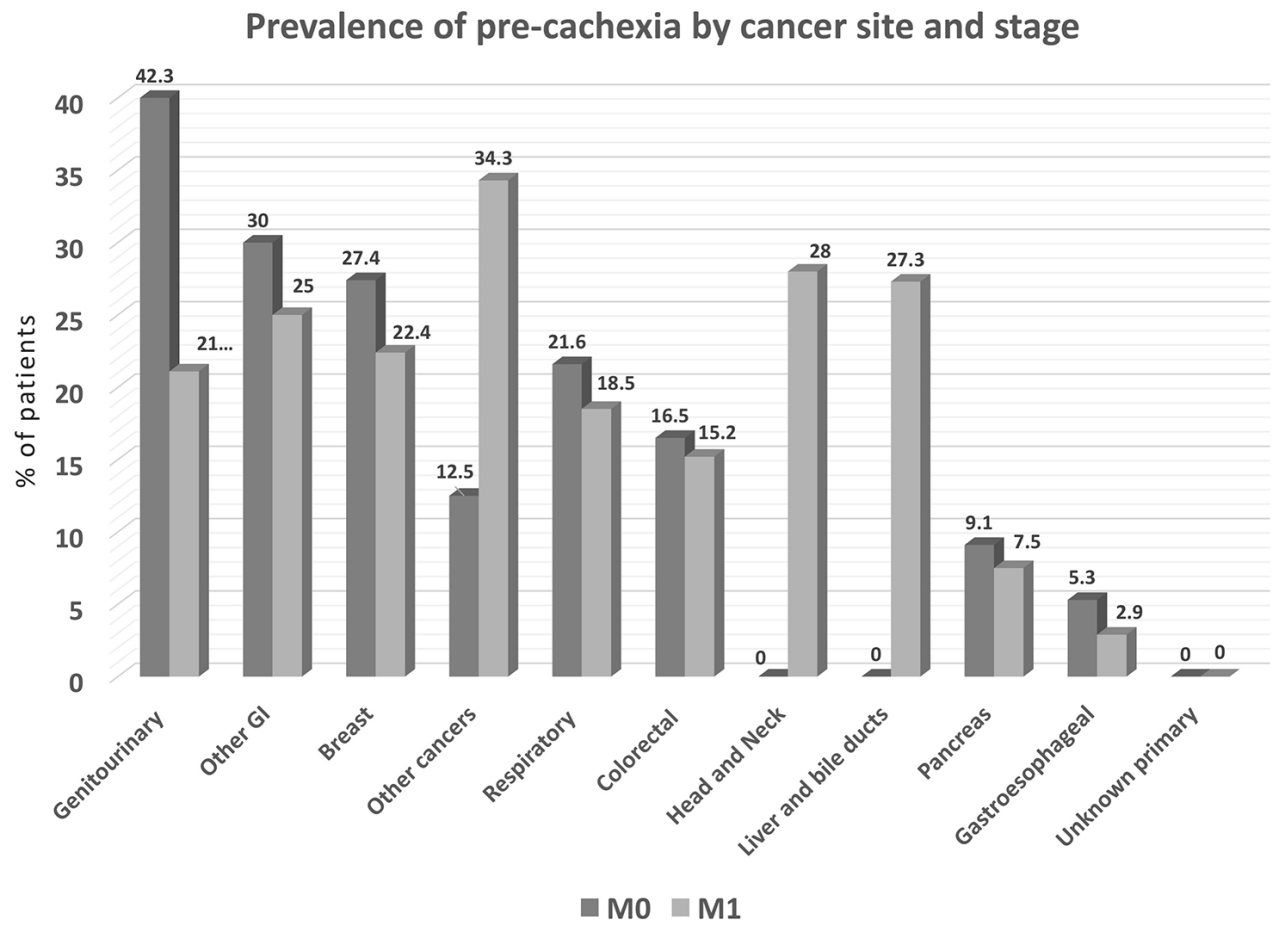
Prevalence of pre-cachexia by cancer site, as determined by percent of patients with unintentional weight loss up to 5% during prior 6 months, along with chronic systemic inflammation and anorexia-related symptoms (*N=*1085) M0 = stage I-III, M1 = stage IV. *P<*0.05 among cancer site groups.

We also assessed for the presence of pre-cachexia in a subset of patients (*N=*1085). Pre-cachexia criteria were cumulatively met by 16.1% of patients (range 3.4% to 28.8% at different primary tumor sites); the prevalence of pre-cachexia in M0 and M1 patients is shown (Figure 5). Cumulatively, the highest prevalence of pre-cachexia was observed in the “other cancers” group. This group includes neoplasms known to have only limited impact on nutritional status, i.e., sarcomas, mesotheliomas, mesenchymal, skin, endocrine and hematologic tumors. As expected, the prevalence of malnutrition and cachexia in this group of patients was relatively low (Figures 4 and 5, respectively).

### Inflammation as revealed by elevated CRP

**Figure 6:**
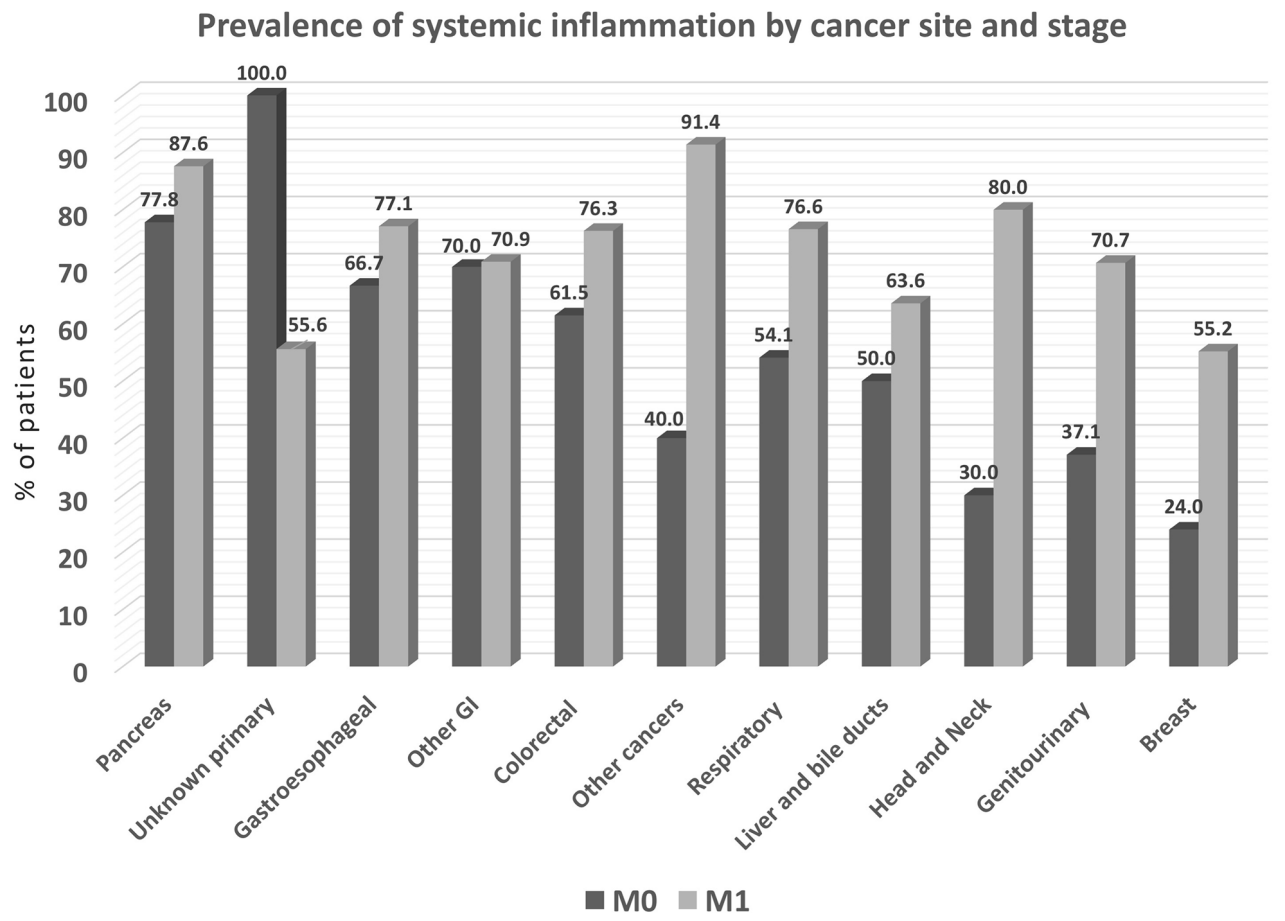
Prevalence of systemic inflammation by cancer site, as determined by % patients with elevated blood levels of C-reactive protein (*N=*1087) M0 = stage I-III, M1 = stage IV. *P<*0.001 among cancer site groups.

Serum C-reactive protein (CRP) determinations were available only in a subset of patients (*N=*1087). Cumulatively, more than 50% of these patients with all tumor types, except breast cancer, had CRP levels greater than the upper normal limit (Figure 6). With the exception of unknown primary tumors, the prevalence of elevated CRP was higher in M1 with respect to M0 patients (*P<*0.001). Further, CRP levels correlated positively with cancer stages (r =0.256, *P<*0.001), the presence of cachexia (r =0.189, *P<*0.001), and weight loss (r =0.232, *P<*0.001), and correlated negatively with anorexia-related scores (r =-0.216, *P<*0.001 for VAS score, r =-0.251, *P<*0.001 for FAACT score) and malnutrition-related scores (r =-0.262, *P<*0.001 for MNA score).

## DISCUSSION

At Italian cancer care centers, oncology physicians enrolled patients in the PreMiO study and assessed nutritional status on their first visit for care. The oncologists reviewed recent weight changes, assessed appetite, looked for evidence of inflammation, and employed validated scoring methods and criteria to detect malnutrition, anorexia, and cachexia. Findings showed that first-visit PreMiO patients were often malnourished or at risk for malnutrition when they entered the study, i.e., 40% to 80% of patients had signs and indicators of nutritional impairment even in early disease stages, particularly in gastroesophageal, pancreatic, head and neck, and colorectal cancer patients. Importantly, our study demonstrated that oncologists can be effectively trained to perform assessments that identify malnutrition and its risks.

Malnutrition is prevalent with cancer, and both percent weight loss and BMI predict survival independently of conventional prognostic factors [26]. Accordingly, newly published expert guidelines advise nutrition screening and assessment for all cancer patients [18]. In the presence of a tumor, the body mounts an intense inflammatory response [27] associated with anorexia and cachexia, which can lead to progressive loss of skeletal muscle mass (with or without loss of fat mass) and worsen impairment of function [27]. The pathophysiology of cancer cachexia is characterized by negative protein and energy balance, which is driven by a variable combination of reduced food intake and abnormal metabolism [14]. Elevated blood CRP, a biochemical marker of inflammation, can be used to help detect cancer-related nutrition problems that predispose to poor outcomes [27]. Systemic inflammation was highly prevalent in PreMiO patients with non-metastatic disease, as well as for those with metastatic disease; based on CRP measures for 56% of study patients, inflammation by cancer site ranged from 21% to 100% of M0 patients and 55% to 91% in M1 patients (Figure 6).

There is no simple biomarker for malnutrition nor is there expert agreement on which screening/assessment tools are most accurate [28]. The MNA has been used successfully for lung cancer patients [29, 30], demonstrating a better predictive and prognostic value compared with measuring weight loss alone in the baseline nutritional evaluation of patients [30]. Because overweight and obesity are widespread today, malnutrition may be overlooked when conventional anthropometric measures such as height, weight, and body mass index are used exclusively for risk assessment [7]. It is therefore important to use screening tools and assessment methods that take other factors into account, e.g., recent weight loss, loss of appetite, loss of lean body mass, and impairment of physical abilities [29, 31, 32]. Specialized screening methods (bioimpedance analysis), and precise tools for assessment of lean body mass (computed tomography) are newly recognized as favorable ways to identify cancer-related malnutrition or its risk [7, 10, 32].

In this study, the prevalence of overt malnutrition (determined by MNA [33]) was considerably lower than the prevalence of cachexia (using Fearon’s criteria;[14] see Figures 3 and 4). This disparity is attributable largely to the different diagnostic criteria used to determine malnutrition and cachexia. Recently published guidelines from the European Society for Enteral and Parenteral Nutrition (ESPEN) [13], classify cachexia of chronic diseases—including cancer—as a form of “disease-related malnutrition (DRM) with inflammation,” and propose that DRM with inflammation and cachexia are interchangeable terms. Nonetheless, while the concepts are aligned, the diagnostic criteria are different. This creates confusion that leads to delays in identifying and treating cancer-related malnutrition and cachexia in everyday clinical practice. We join with others to urgently call for scientific societies to align the definitions and diagnostic criteria of DRM with inflammation and cachexia [28].

Oncologists often question whether preventing or treating cancer-related malnutrition will affect their patients’ prognosis. Recent evidence helps resolve the uncertainty, as available data suggest there are benefits of nutritional intervention to improve outcomes in cancer patients. For example, the high prevalence of malnutrition at diagnosis of gastric cancer was associated with high rates of surgical site infections following surgical tumor removal; the rate of surgical site infection was significantly reduced when patients were given well-managed pre-operative nutrition support [34]. Even for cancer patients who are not malnourished before surgery, 14-day pre-surgical nutrition therapy significantly improved nutritional status and reduced post-operative surgical complications compared to cancer patients who did not receive pre-surgical nutrition support [35]. When lung cancer patients were given high-energy oral nutritional supplements containing eicosapentaenoic acid (a fatty acid with inflammation-blunting properties), food intake and body composition improved, fatigue decreased, and appetite improved, as did measures of physical function and quality of life [36, 37].

Nutritional status affects acceptability and tolerability of anticancer therapies, in turn altering therapeutic choices. An accurate evaluation of nutritional status is of paramount importance in treating cancer patients, especially in early stages [32]. The efficacy of chemotherapy, for example, could be impaired by a reduction in the patient’s therapy tolerance, which is influenced by a poor nutritional status [7, 38].

Lastly, in cancer patients, the relationship between disease curability/severity and subjective symptoms, such as appetite loss or degree of malnutrition, underlines the need for an integrated support team including a psycho-oncologist, who can address and treat psychological aspects (depression, loss of hope, and anxiety) while other team members deal with medical issues.

Altogether, results of the PreMiO study support a call-to-action for oncologists to (1) be aware of malnutrition risk in their patients, even in those with non-metastatic disease, (2) conduct early nutrition screening and make ongoing assessments of nutritional status of cancer patients, and (3) commit to early, aggressive treatment of malnutrition as part of routine supportive cancer treatments. We hope that as nutritional assessment and therapy become routine, survival and quality of life will improve for cancer patients [39].

## MATERIALS AND METHODS

### Study design

PreMIO was a prospective, observational, multicenter study to assess nutritional status and related factors in cancer patients (ClinicalTrials.gov: NCT01622036). The study was conceived, promoted and carried out jointly by the Italian Society of Artificial Nutrition and Metabolism (SINPE) and the Italian Association of Medical Oncology (AIOM); the protocol was published online at https://clinicaltrials.gov/ct2/show/NCT01622036?term=premio&rank=1.

Enrollment was conducted at ESMO (European Society for Medical Oncology)-designated Centers of integrated Oncology and Palliative Care and other medical oncological centers (*N=*22) in Italy.

Inclusion criteria were: patients at first medical oncology visit; diagnosis of solid tumor; age >18 years; no previous anticancer therapies (e.g. radiotherapy or chemotherapy); life expectancy >3 months according to a palliative prognostic score;[40] and informed consent. Cancer type and stage of disease were determined by the oncologist.

Exclusion criteria were inability to feed orally or intestinal obstruction; decompensated metabolic disorders; severe liver failure (total bilirubin >1.5 mg/dL (25μmol/L), and aspartate aminotransferase (AST or SGOT) to alanine aminotransferase (ALT or SGPT) ratio >2-times the Upper Limit Normal (ULN) or, in the case of metastatic liver cancer, >5-times ULN; severe kidney failure indicated by creatinine >2.0 mg/dL (177 μmol/L) or creatinine clearance (ClCr) <50mL/min; acute decompensated heart failure; active infection; primary brain tumor or metastatic brain tumors; severe psychiatric disorders; Mini-Mental State Examination (MMSE) score <25/30 (in patients aged >70);[41] and inadequate logistical support for participation. These criteria eliminated the sickest patients and those least able to consume adequate nutrition, thus creating a higher bar for demonstrating malnutrition prevalence in the remaining study subjects.

### Variables

PreMiO quantified the occurrences of malnutrition and its risk, anorexia and appetite loss, weight loss, and pre-cachexia/cachexia at the first medical oncology visit (Table 3). Patient-perceived disease severity and treatability were also assessed. All evaluations were performed by an oncologist or Senior Resident in Oncology. Prior to the start of the study, these physicians were identified and trained to use the study’s nutrition assessment tools.

**Table 3:** Malnutrition terms and measurements tools

	Anorexia and limited food intake	Pre-cachexia and cachexia	Sarcopenia
**Description**	Food intake falls as a result of: • Altered appetite signals from tumor or its treatment • Physical issues that limit food intake	Weight loss worsens as: • Inflammatory cytokines drive catabolism, increasing metabolic needs • Nutrient intake continues to fall	Cachexia and anorexia can lead to sarcopenia: • Body fat reserves may become depleted • Lean body mass is lost • Physical function declines
**Nutrition tools used in PreMIO study:**			
• **Malnutrition screening**	Mini Nutritional Assessment (MNA)		
• **Nutrition assessment**	VAS – patient-reported intake	CRP above upper limit of normal	
	FAACT – patient perception of signs, symptoms of anorexia/cachexia		
		Weight loss >5% during prior 6 months (or > 2% for patients with low BMI or sarcopenia)	

FAACT, Functional Assessment of Anorexia-Cachexia Therapy (questionnaire); VAS, visual analog scale of appetite; (questionnaire); CRP, C-reactive protein; BMI, body mass index.

### Malnutrition-related score

Malnutrition (as undernutrition) and risk of malnutrition were identified using the Mini Nutritional Assessment (MNA) tool; malnourished individuals had MNA scores <17, those at risk of malnutrition had scores of 17 to 23.5, and well-nourished patients >23.5 [29, 30]. The MNA has been found to correlate with laboratory parameters used to identify inflammation associated with cachexia, and was independently associated with survival in a study of metastatic lung cancer patients (median age 66 years) [29].

#### Anorexia-related score

Appetite loss (anorexia) is commonly evaluated in cancer patients with a two-step questionnaire, first to determine the presence of appetite loss, and next to quantify it. For the first step, this study used a modified version of Anorexia-Cachexia Subscale (AC/S-12) of the Functional Assessment of Anorexia-Cachexia Therapy (FAACT) questionnaire [17, 42]. Next, appetite loss was quantified on a visual analog scale (VAS) of appetite [17]. The FAACT score quantified patient perception of symptoms and signs that correlated with anorexia, and the VAS appetite score represented the patient’s perception of his or her appetite. Patients self-reported oral food intake on a VAS scale of 0 (no food intake) to 100 (normal food intake). The PreMiO study used cutoff points of FAACT score ≤30 for anorexia and VAS ≤70 for appetite loss representative of anorexia [17]. These cutoff values were recently established in a study of patients with advanced cancer evaluated prior to chemotherapy [17].

#### Weight loss

Weight loss was determined as the difference between the patient’s usual weight (6 months ago, as recalled by patient) and his or her weight at study entry. In a recent study, weight loss in cancer patients independently predicted survival, and a gradient of decreasing survival was observed as the percentage of weight loss increased and BMI decreased [26].

#### Pre-cachexia and cachexia determination

Pre-cachexia is a disease-associated condition characterized by unintentional weight loss up to 5% during prior 6 months, along with chronic systemic inflammation and anorexia-related symptoms (as determined by VAS and FAACT results defined above). Inflammation was identified as CRP level above the ULN for the assay used; anorexia-related symptoms (as determined by VAS and FAACT results defined above). Cachexia was identified based on criteria defined by Fearon et al:[14] disease-associated weight loss >5% during the prior 6 months or by the combination of progressive weight loss (more than 2%) and BMI <20 kg/m^2^.

#### Disease severity and curability

The patient-perceived severity and curability of the disease was measured to assess the relationship between patient-perceived degree of severity/curability and psychological well-being, quality of life, or other subjective evaluations. Participants rated severity and curability of their disease on two ten-point scales ranging from 1 (not severe or difficult to cure) to 10 (highly severe or easy to cure). The perception of control and curability has an effect on depression and anxiety [43], which may, in turn, influence nutritional status.

### Data collection

Patient information was recorded on a data collection sheet at the time of enrollment and then uploaded to a dedicated website platform. Patient anonymity was maintained by assigning each patient a study identification number. The data generated at each participating center were compiled at the Coordination Center of the Istituto Dermopatico dell’Immacolata, officially designated by the Ministry of Health as a Centre for Treatment and Research (IRCCS). Patients were stratified by cancer type/site and disease stage, and by age, sex, and general health condition.

### Statistical analyses

All statistical analyses were performed using IBM SPSS Statistics version 20.0 (SPSS Inc., Chicago, IL, USA). Continuous variables were expressed as mean value ± standard deviation (SD). Analysis of variance (ANOVA) was used for analysis of patients’ perceptions of their appetite and the severity and the treatability of their condition. The chi-squared test was used to evaluate the prevalence of unintentional weight loss in different groups. The Spearman correlation test was used to determine the strength of correlations between cancer site, inflammation, and perception of anorexia [44].

### Ethical considerations

Data were collected under the auspices of research ethics approvals for human experimentation at all contributing institutions and at the IDI-IRCCS.

## SUPPLEMENTARY MATERIALS TABLES



## Abbreviations

AC/S-12: Anorexia-Cachexia Subscale
BMI: Body mass index
CRP: C-reactive protein
FAACT: Functional Assessment of Anorexia-Cachexia Therapy
M0: Localized cancer, stages I-III
M1: Metastatic cancer, stage IV
MNA: Mini Nutritional Assessment
VAS: Visual analog scale of appetite
ULN: Upper Limit Normal
