# Supplementary material for: Prevalence of malnutrition in patients at first medical oncology visit: the PreMiO study
Source: Oncotarget. 2017 Aug 10;8(45):79884–96. doi: 10.18632/oncotarget.20168 (PMC5668103; doi:10.18632/oncotarget.20168)
Supplement: Supplementary file 1 [file oncotarget-08-79884-s001.pdf]

Prevalence of malnutrition in patients at first medical oncology visit: the PreMiO study

SUPPLEMENTARY MATERIALS

Supplementary Table 1: Patient-perceived disease severity by cancer site, based on VAS scores (*N*=1857)

Cancer site	VAS disease severity M0	VAS disease severity M1	VAS disease severity Total
**Breast**	5.5 ±3.2	7.0 ±2.1	5.7 ±3.0
**Genitourinary tract**	5.8 ±3.1	5.8 ±3.0	5.6 ±3.1
**Colorectal**	4.8 ±3.2	5.9 ±2.7	5.3 ±3.0
**Lung**	5.4 ±2.9	6.9 ±2.5	6.5 ±2.7
**Other cancer[1]**	5.4 ±3.0	6.7 ±2.3	5.6 ±2.9
**Gastroesophageal**	5.3 ±3.3	6.0 ±2.9	5.7 ±3.0
**Pancreatic**	5.2 ±3.6	7.1 ±2.6	6.4 ±3.1
**Other GI**	6.6 ±3.1	5.4 ±3.1	5.3 ±3.4
**Liver/bile duct**	3.4 ±3.8	5.6 ±2.3	5.2 ±2.7
**Head and neck**	4.6 ±3.0	6.8 ±2.1	5.9 ±2.6
**Unknown primary site[2]**	7.0	5.4 ±2.5	4.0 ±3.2
**ALL CANCERS**	5.4 ±3.2	6.4 ±2.7	5.7 ±3.0

Mean patient-perceived severity score was 5.7 ±3.0 and patient-perceived curability was 6.0 ±3.1. Most of the of patients who perceived their disease as severe (score ≥6, N=983, 52.9%) as opposed to not severe (score <6, N=874, 47.1%) had breast (20.2% and 21.3%), lung (19.0% and 14.4%), genitourinary (16.3% and 16.5%) and colorectal cancer (15.3% and 19.1), (P=0.001), and had metastases (59.3% vs 39.8%), P<0.001.

[1]Other cancer includes: Sarcoma, Mesothelioma, Mesenchymal, Skin, Endocrine and Hematologic tumors.

[2] *N*=1 if SD not indicated

VAS, Visual analog scale of appetite (questionnaire); SD, standard deviation.

Cutoff points for the perception of higher disease severity is VAS ≥6. M0 = stage I-III, M1 = stage IV. Data are expressed as Mean ±SD.

Supplementary Table 2: Patient perceived disease curability by cancer site, based on VAS scores (*N*=1857)

Cancer site	VAS disease curability M0	VAS disease curability M1	VAS disease curability total
**Breast**	6.7 ±3.4	6.6 ±2.2	6.4 ±3.2
**Genitourinary tract**	6.8 ±3.2	5.8 ±3.1	6.0 ±3.3
**Colorectal**	5.6 ±3.6	6.1 ±2.8	5.8 ±3.2
**Lung**	5.5 ±2.9	6.3 ±2.4	6.1 ±2.5
**Other cancer[1]**	7.3 ±3.1	6.1 ±2.4	6.0 ±3.1
**Gastroesophageal**	5.8 ±3.3	5.6 ±2.7	5.6 ±2.9
**Pancreatic**	4.3 ±3.3	6.3 ±2.3	5.6 ±2.8
**Other GI**	7.0 ±3.3	5.1 ±3.1	5.3 ±3.5
**Liver/bile duct**	5.2 ±4.8	5.8 ±2.0	5.5 ±2.6
**Head and neck**	5.9 ±3.5	6.7 ±2.2	6.2 ±2.7
**Unknown primary site[2]**	5.0	4.8 ±2.1	3.5 ±2.8
**ALL CANCERS**	6.3 ±3.4	6.1 ±2.6	6.0 ±3.1

Most of the of patients who perceived their disease as difficult to cure or incurable (<6, N=856; 46%) as opposed to those who perceived their disease as curable (≥6, N=1001; 54%) had breast (18.3% and 23%), colorectal (17% and 17%), lung (16.6% and 17%) and genitourinary (15.7% and 17%) cancer (P=0.009), and had metastasis in 52% vs 48% of cases, P<0.001.
[1]Other cancer includes: sarcoma, mesothelioma, mesenchymal, skin, endocrine and hematologic tumors.

[2]N1 if SD not indicated.

VAS, Visual analog scale of appetite (questionnaire); SD, standard deviation

Cutoff points for the perception of poor disease curability is VAS <6. M0 = stage I-III, M1 = stage IV. Data are expressed as Mean ± SD.

Supplementary Table 3: VAS appetite, FAACT and MNA score according to patient perception of disease severity and curability

	Not severe / easy to cure (*N*=273)	Severe/easy to cure (*N* =728)	Not severe /difficult to cure (*N*=601)	Severe/difficult to cure (*N*=255)	*P* value**
VAS appetite	69 ±21	69 ±21	68 ±24	56 ±23	<0.001
FAACT	31 ±6	30 ±6	31 ±6	27 ±6	<0.001
MNA	24 ±4	23 ±4	24 ±4	21 ±5	<0.001

Disease was judged by patients as “not severe and easy to cure” in 273 cases (14.7%), “not severe and difficult to cure” in 601 (32.4%), “severe/easy to cure” in 728 (39.2%), and “severe/difficult to cure” in 255 (13.7%).
*Cutoff values: FAACT score ≤30 for anorexia; VAS score ≤70 for appetite loss representative of anorexia; MNA scores: malnourished <17; at risk of malnutrition, 17 to 23.5; well-nourished >23.5.

**ANOVA univariate test.

VAS, Visual analog scale of appetite FAACT, Functional Assessment of Anorexia-Cachexia Therapy (questionnaire); MNA, Mini Nutritional Assessment; SD, standard deviation

Data are expressed as Mean ± SD*.
